# Remarks on the Pathology If Iritis

**Published:** 1845-01

**Authors:** 


### Remarks on the Pathology of Iritis. By *J. F. France.* (Guy's Hospital Reports, October, 1844.)

Simple iritis, if it ever occur independently of injury, is a rare disease; and the author's remarks are directed to the consideration of the syphilitic and arthritic forms, especially the former, which is by far the most frequent of all. The early and middle periods of manhood are the ages most liable to syphilitic, while elderly persons are more often the subjects of arthritic iritis. In both, the feeble and cachectic are those who chiefly suffer, at least among the applicants at hospitals. The appearance of the iris in the two cases is very different. In *syphilitic* iritis the mobility and the brilliancy of the iris are soon diminished; and before long, at one or more points, its structure becomes tumefied, and its colour changed to a reddish brown. More or less exudation from the aqueous membrane attaches portions of the edges of the pupil to the capsule of the lens. The quantity of fibrin effused into the anterior chamber may be very great. It is, however, in the *arthritic* form of the disease that the lining of the anterior chamber, and serous covering of the iris, are chiefly affected. A more extensive and considerable dulness is present, and copious fibrinous effusion rapidly ensues. The *entire* pupillary margin soon becomes fixed, and even the whole area of the pupil may be overspread with a white or yellowish effusion. The iris is not partially discolored in rusty-looking spots, as in the syphilitic form, but becomes uniformly changed to a greenish or yellowish colour. If there is tumefaction it is also general. In the one disease the serous coverings of the iris are especially affected, in the other its parenchymatous structure.

The tuberculated and discolored condition of the iris have been erroneously attributed to depositions of lymph on the free surface of the iris; but, if the eye be closely and repeatedly examined, the tumefaction will be found to arise from the swollen state of the membrane itself, caused by interstitial deposition. The reddish colour is produced by the ramification of vessels, containing red blood, in the aqueous membrane over the inflamed points. The dilatation of these minute vessels must reach a certain point before this cause of the red colour can be detected.

"In moderately severe cases this soon takes place; and, if then, the eye be inspected closely in a good light, and with the assistance of a powerful lens (the cornea and aqueous membrane being clear, and photophobia not great) sometimes only one or two, but occasionally a complete net-work of vessels become manifest, ramifying over the swollen parts of the iris, and evidently imparting to them the rusty red colour which they exhibit. The appearances I now describe may be detected in so large a proportion of cases where this dusky colour is developed, that I cannot doubt that the same hue arises from the same cause, when, from the mildness of the attack, and the consequent slighter vascular distention, the individual vessels elude observation; or intolerance of light forbids that close examination and exposure of the globe which are necessary, in most cases, for their recognition. In some rare cases these vessels are so gorged, crossing over a well-grown tubercle, that the naked eye can scarcely fail to notice them: in such instances they have in fact been observed, and been ascribed to organization having taken place in lymph thrown out into the anterior chamber from the free surface of the iris."

Eighteen cases are adduced in illustration of the various points laid down; and the author concludes a very good paper with some judicious remarks upon the *treatment*. Among the patients at Guy's general bleeding is never, or very rarely, resorted to, in *syphilitic* iritis, an effectual impression being usually made with facility by means of cupping and purging. After this, mercury is given, in such doses and forms as may seem most applicable to the case, the object being to affect the mouth, or cause recession of the disease. Oftentimes, in the cachectic, sarsaparilla and the iodide of potassium must be given even during the mercurial course. Hyosciamus is preferable, as a combination with mercury, to opium, which favours contraction of the pupil, adding chalk if purging comes on. Mild doses of the mercury are required long after the more active symptoms have subsided, and are then compatible with tonics and improved diet. Blisters are useful when cupping can be carried no farther; and the severe pain in the region of the temple is much relieved by the application of the ung. hyd. c̄ opio. there. Turpentine has not been found useful by the author, and he has been obliged to discontinue it in some cases, owing to its irritating effects upon the kidneys.

In *arthritic* iritis, less vigorous measures are called for. Very moderate cupping, alterative doses of mercury, accompanied by salines and colchicum, are the principal remedies, these last being soon replaced by tonics and alkalis. Extractum conii (gr. v.—x. ter.) often exerts a rapidly beneficial influence upon this disease, even when the inflammation has been very serious. The application of belladonna occasions great suffering in this form of the malady.

A venereal iritis follows occasionally *gonorrhœa*, and then puts on all the characters of arthritic iritis.

